# A rare lumpy inflammatory lesion of the orbit: a unique case report

**DOI:** 10.1186/s13000-023-01316-w

**Published:** 2023-02-23

**Authors:** Rui Liu, Jing Li, Tingting Ren, Hong Zhang, Jianmin Ma

**Affiliations:** 1 Beijing Institute of Ophthalmology, Beijing Tongren Eye Center, Beijing Tongren Hospital, Capital Medical University, Beijing, 100730 China; 2 Pathology Department, Beijing Tongren Hospital, Capital Medical University, Beijing, 100730 China

**Keywords:** Eosinophilic angiocentric fibrosis, IgG4-related disease, Histopathological examination, Diagnosis, Case report

## Abstract

**Background:**

Eosinophilic angiocentric fibrosis (EAF) is a rare inflammatory lesion, especially in orbit. EAF is believed to be related to IgG4-related disease (IgG4-RD), but the clinical manifestations of systemic involvement are relatively rare and easy to be confused with tumors or other inflammatory diseases. Histopathological examination is the most important way of its diagnosis and differentiation.

**Case presentation:**

We presented a 55-year-old female patient presented with recurrent swelling of the right lower eyelid for more than 2 months. The pathological diagnosis was EAF. Positive immunostaining for IgG, CD34, κ, and λ, while negative immunostaining for IgG4.

**Conclusions:**

Complete surgical resection is the preferred treatment, histopathological examination is the main diagnostic standard.

## Introduction

Eosinophilic angiocentric fibrosis (EAF) is a rare and slow progressive inflammatory disease. The main sites of involvement are the nasal cavity and sinuses, although rare cases can also occur in the orbit, upper respiratory tract, gingiva, and brain [1–4]. The etiology and pathogenesis of this disease are still unclear, and its clinical manifestations are nonspecific and diverse, making it easy to confuse with orbital inflammatory lesions or neoplastic lesions in diagnosis. In this paper, we report a case of orbital EAF and summarize the clinical characteristics of the disease based on the literature to provide a reference for clinical diagnosis and treatment.

## Case presentation

**Fig. 1:**
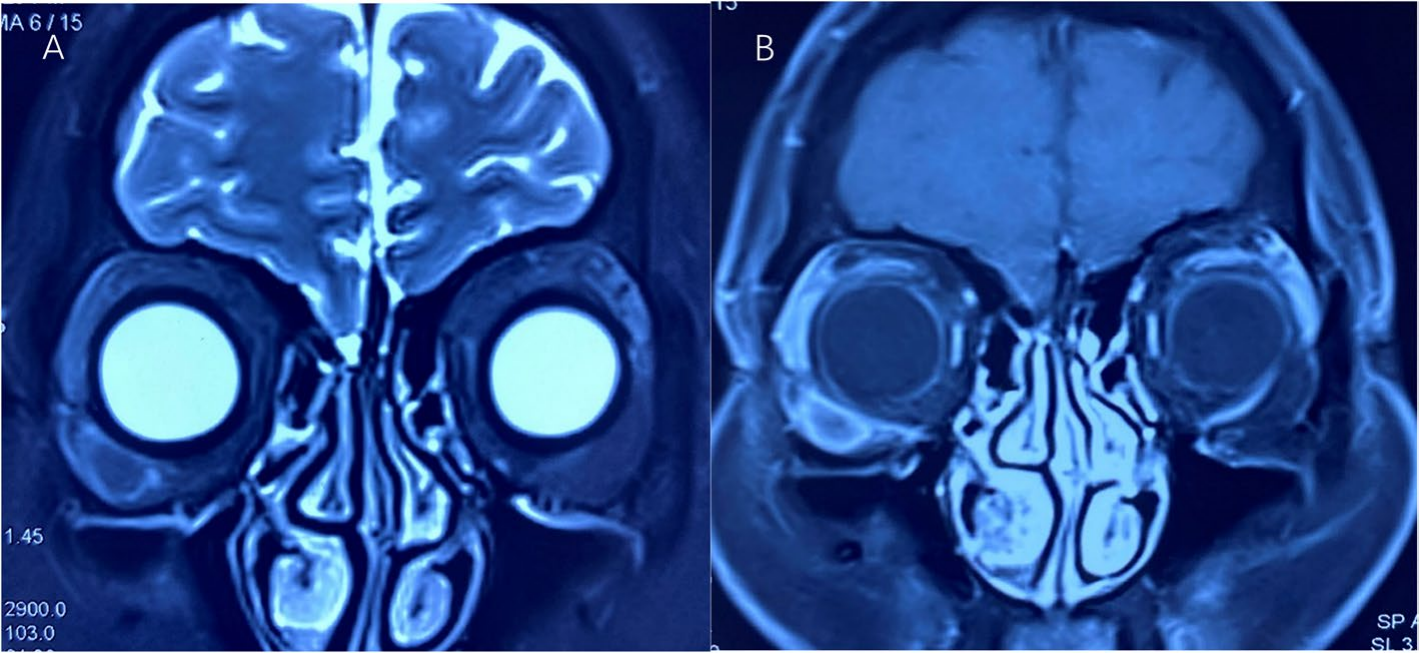
MRI images of the orbit. **A** Orbital T2-weighted image (T2WI) shows clear tumor boundaries and slightly short T2 signal. **B** Enhanced orbital MRI scan shows significant enhancement of the tumor

**Fig. 2:**
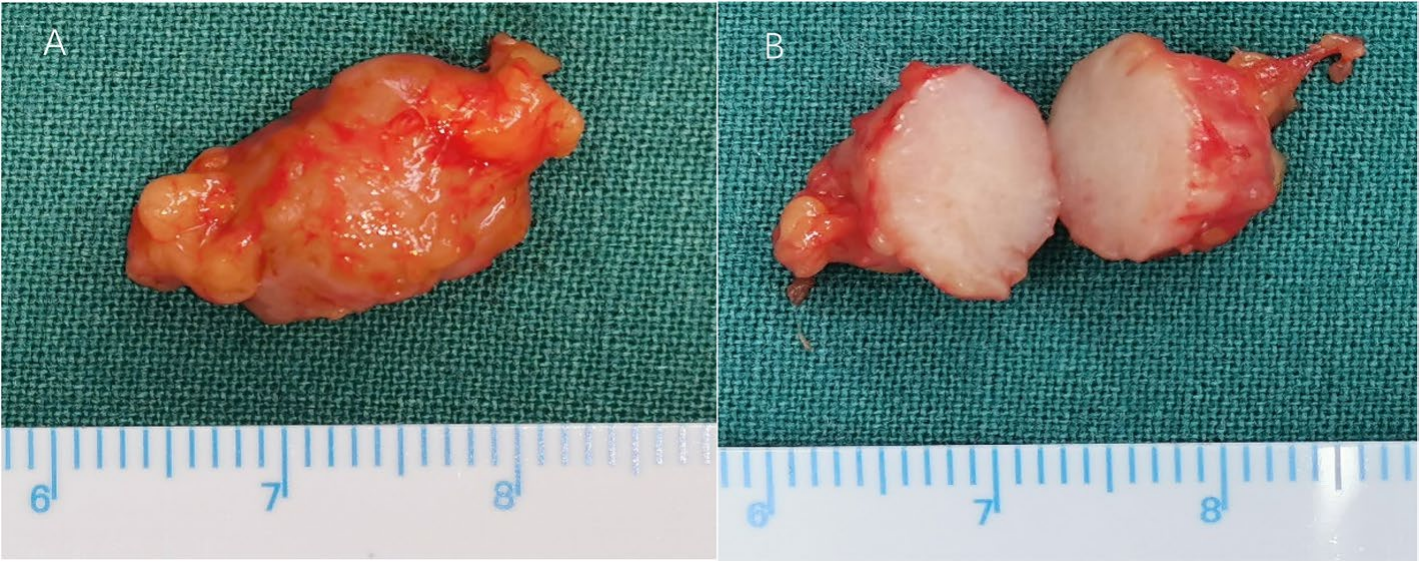
General appearance of the mass. **A** Clear boundary. **B** Profile

A 55-year-old female patient presented with recurrent swelling of the right lower eyelid for more than 2 months. She had a history of hypertension and hysterectomy. Ophthalmic examination showed binocular visual acuity of 1.0, normal intraocular pressure, and normal anterior ganglium and fundus. The tumor, which was tough, painless, and movable, was palpable near the zygomatic arch of the lower right eyelid. Laboratory examination showed the patient to be negative for syphilis antibody. Orbital magnetic resonance imaging (MRI) showed oval-like T1 and slightly shorter T2 signal shadows in the lower quadrant of the right outer orbit, with clear boundaries and significantly enhanced annular edges (Fig. 1A, B). To clarify the nature of the lesion, we removed the right orbital mass under general anesthesia with the patient’s consent. The tumor was completely dissected during surgery. It was 1.6 × 1.0 × 1.0 cm in size, gray, and homogeneous, with a clear boundary (Fig. 2A, B).

## Histopathology

**Fig. 3:**
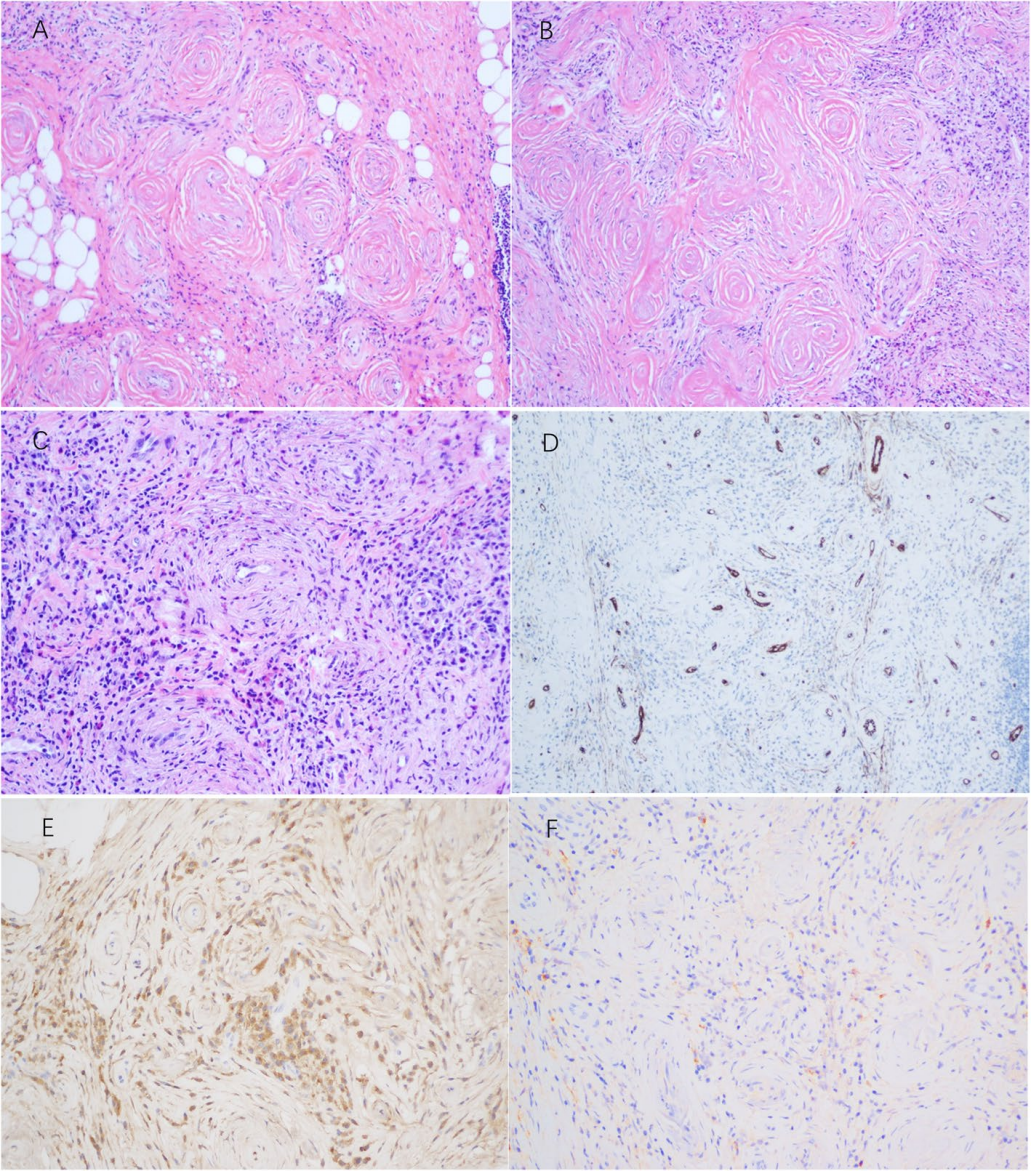
Histopathological results for the lesion. **A** Fibrous-tissue hyperplasia is present, and the vascular wall shows scallion skin–like fibrotic changes. **B** Some of the fibrotic areas may be joined. **C** Additional infiltration of lymphocytes, plasma cells, and eosinophils in and around the vascular wall. **D** Positive expression of CD34 outlines the vascular endothelium. **E** Positive expression of IgG. **F** Negative expression of IgG4

Histopathological examination of the resected mass showed infiltration of lymphocytes, plasma cells, and eosinophils in and around the vascular wall; fibrous-tissue hyperplasia; and fibrotic vascular-wall changes like onionskin, consistent with eosinophilic vascular-center fibrosis (Fig. 3A-F). Immunochemical (IHC) results were as follows: negative for immunoglobulin G4 (IgG4) and positive for IgG, CD34, κ, and λ. The patient took oral methylprednisolone after surgery, and the dosage was gradually reduced over the subsequent 3 months. Postoperative recovery was good, and no tumor recurrence was observed during 1-year follow-up.

## Discussion

Orbital EAF progresses slowly. Its various clinical manifestations include diffuse inflammatory lesions or localized mass lesions, causing it to be easily misdiagnosed as other diseases. It is also invasive, spreading easily to surrounding tissues and often causing bone damage, making timely diagnosis and treatment very necessary.

With this in mind, we collected a total of 18 cases of orbital EAF via a literature search and combined them with the above-described case for analysis to summarize the clinical characteristics of the disease [5–18]. Of the 18 cases, 6 involved naso-orbital–communication lesions and 12 involved localized orbital lesions (Table 1). Average age of onset was 47.67 ± 12.70 years. There were nine males and nine females for a male: female ratio of 1:1, indicating no significant gender difference. However, some studies have shown that women are more prone to EAF [11, 19]. The disease is commonly monocular in incidence, but binocular cases can also occur; the monocular: binocular ratio is 7:1. In 12 cases (66.7%), lesions were located in the orbit; in 2 (11.1%), they were in the lacrimal gland; and in 1 case each (5.6%), they were in the eyelid, conjunctiva, epicanthus and rectus. The main clinical manifestations were eyelid, orbital, or conjunctival swelling in ten cases (66.7%); eyeball protrusion in five (33.3%); tearing, eyeball displacement, and headache in three (20.0%); and eye pain, limited eye movement, and vision loss in two (13.3%). Nasal congestion, nasal bleeding, loss of smell and other symptoms can also be associated with naso-orbital communication lesions. Studies have shown that nasal congestion is the most common symptom of nasal EAF, appearing in about 66.7% of cases [1]. Six cases had extraocular-muscle involvement, mostly of the internal rectus muscle. Six cases involved periorbital soft tissue, five involved bone destruction, two involved the optic nerve, and one involved the sclera.

**Table 1:** Clinical features of 17 cases of orbital EAF

Case	Age	Gender	Laterality	Position	Clinical features	Destruction	Serum IgG4	Tissue IgG4	ANCA	Treatment	Prognosis	Follow-up
Our case	55	Female	Right	Orbital mass	Eyelid swelling	None	Negative	Negative	Negative	Surgery+glucocorticoid	No recurrence	2 months
Heedari [5]	69	Male	Left	Medial orbital mass	Periorbital edema, nonaxial proptosis and lateral globe displacement, limited abduction	Medial orbital wall, inferior rectus, medial rectus muscles	Negative	Positive, 40–45%	Negative	Surgery+glucocorticoid	No recurrence	4 months
Mansfield [6]	37	Female	Left	Lateral orbital mass	Periorbital swelling	Scleral thickening	Negative	Positive, < 10%	Negative	Biopsy+ glucocorticoids+ rituximab+ methotrexate / azathioprine	Multiple recurrence	18 months
Okuyama [7]	55	Female	Bilateral	Upper eyelid conjunctivas	Conjunctival swelling	None	179 mg/dl	Positive, 45%	–	Surgery	No recurrence	6 months
Legare [8]	58	Male	Left	Orbit and sinuses illustrated a solid mass	Eyelid swelling	Nasal soft tissue, medial periorbital soft tissue, bony nasal destruction, nasal septum and anterior nasal cavity mucosa	–	Positive	Negative	Surgery+glucocorticoid/ rituximab	Remission	6 months
Gorostis [9]	61	Male	Right	Ethmoido-orbital mass	Visual loss, pain, proptosis, eyelid oedema, headache, nasal obstruction	Ipsilateral ethmoidal air cells and optic nerve, the periorbital fat, osteolysis of the lamina papyracea, medial oculomotor muscles	> 135 mg/dl	Positive,> 40%	–	Surgery+glucocorticoid/dapsone, Surgery+glucocorticoid/ rituximab	Recurrence (5 years later), remission	6 years
Chen [10]	32	Female	Left	Upper eyelid lesion	Bleeding, itching, clear discharge, headaches	Bilateral preseptal soft tissue swelling, maxillary sinuses, nasal cavity	Negative	Positive，10%	Negative	Surgery+glucocorticoid/ rituximab	Remission	6 months
Faramarzi [11]	35	Male	Left	Medial canthal region mass, maxillary sinus mass	Progressive orbital swelling ，left epiphora, proptosis, anterolateral globe displacement, nasal obstruction	Lamina papyracea	–	–	–	Septoplasty, left uncinectomy, middle meatal antrostomy, and anterior ethmoidectomy	No recurrence	12 months
Radhakrishnan [12]	38	Female	Right	Inferior orbital mass	Limited abduction	Lateral rectus, inferior oblique muscles	–	–	Negative	Surgery+glucocorticoid	No recurrence	3 months
Takahashi [13]	43	Male	Right	Medial rectus muscle lesion	Visual loss, eyelid swelling	Medial rectus muscle and optic nerve with soft tissue swelling of the ethmoid sinus	Negative	Positive	Negative	Surgery+intravenous methylprednisolone and oral diaminophenyl sulfone，cyclophosphamide	No recurrence	18 months
Karligkiotis [14]	46	Male	Right	Orbital lesion	Proptosis, lateral globe displacement, right unilateral nasal obstruction, headache	Homolateral ethmoidal cells with oval configuration, right middle meatus, the ethmoid sinus	–	–	Negative	Surgery	Remission	36 months
Deshpande [15]	63	Male	Bilateral	Nasal and lacrimal glands, lung	Ocular tearing, nasal congestion, anosmia	Nose, middle turbinates	1490 mg/dl	Positive,80%	Negative	Surgery+glucocorticoid	–	–
Deshpande [15]	31	Female	–	Orbit, maxillary sinus, ethmoid, nasal	–	–	–	Negative	–	–	–	–
Deshpande [15]	54	Female	Right	Lacrimal gland	–	–	–	Positive,97%	–	–	–	–
Deshpande [15]	55	Male	–	Orbit	–	–	–	Positive, 68%	Negative	–	–	–
Kiratli [16]	30	Female	Right	Inferomedial orbit	Ipsilateral epiphora	Anterior ethmoid cells and right middle turbinate	–	–	Negative	Fluorocortolon+ desloratadine+ Steroid	No recurrence	32 months
Azam [17]	35	Female	Right	Retrobulbar area of right orbit encasing the eyeball	Pain and right lower eyelid swelling	Subcutaneous fat	–	–	Negative	Surgery	No recurrence	8 months
Leibovitch [18]	61	Male	Right	Medial orbit	Periorbital edema and painless proptosis	Fat, medial orbital wall and middle ethmoidal cells	–	–	Negative	Oral steroids	No remission	6 months

“-” stands for unknown

EAF is believed to be a manifestation of IgG4-related disease (IgG4-RD) [3]. Chew et al. showed that about 30% of EAF meets the IgG4-RD standard of the 2019 European League Against Rheumatism (EULAR)/American College of Rheumatology (ACR) classification scheme [4]. Of the 18 cases of EAF we reviewed, 3 had increased serum IgG4 levels, 5 had normal serum IgG4 levels, and serum IgG4 levels in the remaining 10 were unknown. Expression of IgG4 in tissues was increased in 10 cases, with the ratio of IgG4: IgG as high as 80%; it was normal in 2 cases and unknown in the remaining 6 cases. Antineutrophil cytoplasmic antibody (ANCA) was negative in 13 cases. We can therefore see that not all EAF is accompanied by increased IgG4 expression and local lesions are more common. In our case, IHC staining showed negative IgG4 expression, lesions were confined to the orbit, and no other parts of the eye or other bodily organs were involved. None of these findings supported a diagnosis of IgG4-RD.

Histopathological examination is the main standard for diagnosing EAF. The typical pathological manifestations are massive infiltration of eosinophils, plasma cells, lymphocytes, and fibroblasts around small blood vessels; thickening of matrix; and characteristic rotation of onionskin-like collagen fibers and mesh proteins around occlusive blood vessels, accompanied by positive expression of IgG4 and IgG [1, 2]. EAF should be distinguished from orbital inflammatory pseudotumor, hemangioma, and granulomatous disease.

Complete surgical resection is the most important treatment method for this disease. Surgery combined with glucocorticoid therapy has a certain but not strong effect [5]. Rituximab is the most commonly used alternative or adjuvant drug when the patient does not respond to glucocorticoid therapy or when the side effects are relatively serious. Methotrexate and azathioprine are also used. However, when the degree of fibrosis is relatively severe, drugs seem to be ineffective [11]. Localized lesions have better prognosis and less recurrence. Diffuse inflammatory lesions involving the orbit or sinus take longer to treat, recur more frequently, and often require multiple operations. Studies have shown that 62% of sinus EAF cases undergo complete resection, with a recurrence rate of 20% [20]. Therefore, this disease requires close follow-up and observation and timely evaluation of recurrence. We followed up on our patient for 1 year without observing recurrence.

In conclusion, in this paper we report a case of localized EAF and summarize the current literature on this disease. In clinical diagnosis, EAF should be considered when a patient presents with an orbital mass. Complete surgical resection is the preferred treatment, histopathological examination is the main diagnostic standard, and close follow-up is very necessary. The relationship between EAF and IgG4-RD should be elucidated through additional case studies.

## Data Availability

Not applicable.

## Abbreviations

EAF: Eosinophilic angiocentric fibrosis
MRI: Magnetic resonance imaging
IHC: Immunohistochemical
IgG4-RD: Immunoglobulin G4 related disease

## References

[1] Javadirad E, Roozbahani NE, Sadafi S (2022). Eosinophilic angiocentric fibrosis of the sinonasal tract: a case report and review of the literature. J Int Med Res.

[2] Daneshi SA, Taheri M, Fattahi A, Fadavi P (2022). The primary brain eosinophilic angiocentric fibrosis, a rare case report. Prague Med Rep.

[3] Saenz-Ibarra B, Ceceñas-Falcon LA, Cardenas-De la Garza JA, Garza-Elizondo MA, De Hoyos R, Dieste M, Barboza-Quintana O (2020). Nasal eosinophilic angiocentric fibrosis with IgG4-positive plasma cell infiltration. Malays J Pathol.

[4] Chew EJC, Lee MH, Chung HW, Tang PY (2022). Eosinophilic angiocentric fibrosis and immunoglobulin 4-related disease revisited. Histopathology.

[5] Heedari HM, Ciotoracu AC, Mitulescu TC, Dimancescu MG, Enache S, Predețeanu D (2021). A clinical case of orbital inflammatory pseudotumor as the primary expression of eosinophilic angiocentric fibrosis. Rom J Ophthalmol.

[6] Mansfield Smith SC, Clare G, Jones RB (2021). Pregnancy following rituximab for orbital eosinophilic angiocentric fibrosis. Rheumatology (Oxford).

[7] Okuyama S, Yazu H, Ito Y, Minato H, Fujishima H (2020). Eosinophilic angiocentric fibrosis in bilateral upper eyelid conjunctivas: a first case report. Am J Case Rep.

[8] Legare N, Frosh S, Vasquez JB, Ho ST (2018). Eosinophilic angiocentric fibrosis: a sino-orbital masquerader. BMJ Case Rep.

[9] Gorostis S, Bacha M, Gravier S, Raguin T (2017). Right ethmoid eosinophilic angiocentric fibrosis with orbital extension. Eur Ann Otorhinolaryngol Head Neck Dis.

[10] Chen VH, Grossniklaus HE, DelGaudio JM, Kim HJ (2017). A concomitant case of orbital granuloma Faciale and eosinophilic angiocentric fibrosis. Ophthalmic Plast Reconstr Surg.

[11] Faramarzi M, Dadgarnia MH, Moghimi M, Sharouny H, Behniafard N (2015). Nasal eosinophilic angiocentric fibrosis with orbital extension. Head Neck Pathol.

[12] Radhakrishnan S, Adulkar NG, Rangarajan V (2015). Eosinophilic angiocentric fibrosis of the orbit: a case report and review of literature. Indian J Pathol Microbiol.

[13] Takahashi Y, Takahashi E, Ichinose A, Kakizaki H (2015). Orbital compartment syndrome in eosinophilic angiocentric fibrosis. Ophthalmic Plast Reconstr Surg.

[14] Karligkiotis A, Volpi L, Ferreli F, Cerati M, Kagkelari E, Meloni F, Castelnuovo P (2014). Primary orbital eosinophilic angiocentric fibrosis with intranasal extension. Head Neck.

[15] Deshpande V, Khosroshahi A, Nielsen GP, Hamilos DL, Stone JH (2011). Eosinophilic angiocentric fibrosis is a form of IgG4-related systemic disease. Am J Surg Pathol.

[16] Kiratli H, Onder S, Yildiz S, Ozşeker H (2008). Eosinophilic angiocentric fibrosis of the orbit. Clin Exp Ophthalmol.

[17] Azam M, Husen YA, Hasan SH (2010). Eosinophilic angiocentric fibrosis of orbit. Indian J Pathol Microbiol.

[18] Leibovitch I, James CL, Wormald PJ, Selva D (2006). Orbital eosinophilic angiocentric fibrosis case report and review of the literature. Ophthalmology.

[19] Tabaee A, Zadeh MH, Proytcheva M, LaBruna A (2003). Eosinophilic angiocentric fibrosis. J Laryngol Otol.

[20] Heft Neal ME, Rowan NR, Willson TJ (2017). A case report and systematic review of eosinophilic angiocentric fibrosis of the paranasal sinuses. Ann Otol Rhinol Laryngol.

